# Human Rickettsialpox, Southeastern Mexico

**DOI:** 10.3201/eid1510.081507

**Published:** 2009-10

**Authors:** Jorge E. Zavala-Castro, Jorge E. Zavala-Velázquez, Gaspar F. Peniche-Lara, Justo E. Sulú Uicab

**Affiliations:** Human Rickettsialpox, Southeastern Mexico; Universidad Autónoma de Yucatán, Mérida, México

**Keywords:** Rickettsia akari, human cases, molecular diagnosis, rickettsialpox, Mexico, bacteria, rickettsia, dispatch

## Abstract

The detection of *Rickettsia akari* in 2 human patients increased the diversity of rickettsioses affecting the public health in the southeast of Mexico. Rickettsialpox should be considered in the differential diagnosis with other febrile illnesses for the correct diagnosis and accurate treatment of this potential threat to human health.

Rickettsialpox is an illness characterized by fever, headache, papulovesicular rash over the trunk and extremities, and, in 80% of cases, appearance of an eschar. *Rickettsia akari*, the etiologic agent of rickettsialpox, is commonly transmitted by the bite of the house-mouse mite, *Liponyssoides sanguineus*. Human cases of rickettsialpox, as well as infected mites and potential reservoirs of *R. akari*, have been found in several countries, including the United States, Turkey, Croatia, and Ukraine (*1*–*5*). Despite the presence of the house mouse (*Mus musculus*) around the world, in Latin America human cases caused by *R. akari* have not been reported, and rickettsial diseases caused by antigenically related rickettsiae have been confined to *R. rickettsii*, *R. felis*, *R. prowasekii*, *R. typhi*, and *R. parkeri* (*6**–**11*). We report 2 human cases of *R. akari* infection in the Yucatan Peninsula of Mexico.

## The Study

Patient 1 was a 9-year-old girl who came to the public hospital in Merida, Yucatan, in May 2008. Her illness had started abruptly with high fever and headache, then evolved over a 12-day period to include nausea, vomiting, hemorrhagic conjunctivitis, excessive lacrimation, and epistaxis. She was treated empirically with antipyretic drugs and had a slight improvement; 3 days after beginning treatment, fever and epistaxis returned with myalgia; irritability; papulovesicular rash involving the extremities, thorax, and oral mucosa; vaginal and gingival bleeding; and disseminated ecchymoses. Clinical laboratory studies showed hemoglobin 9.9 g/dL and hematocrit 29.0% (reference ranges 12–18 g/dL and 31%–51%, respectively), thrombocytopenia (45 × 10^3^ platelets/mL [reference range 140–440 × 10^3^ platelets/mL]), prolonged prothrombin and thromboplastine times (20 s and 64 s [reference range 10–15 s and 25–35 s, respectively]), neutrophilia, and elevated transaminase (aspartate transaminase 100 mU/mL [reference range 14–36 mU/mL], alanine transaminase 148 mU/mL [reference range 9–52 mU/mL]). The girl was hospitalized in the intensive care unit with a preliminary diagnosis of shock from dengue hemorrhagic fever.

Patient 2 was a 32-year-old woman in whom rickettsialpox was diagnosed in July 2008. She reported visiting a suburban area and being bitten by an unidentified arthropod. Her illness started abruptly with fever, headache, myalgia, and arthralgia in her extremities. The patient showed signs of dengue fever and was treated symptomatically. Three days after the first symptoms, a papulovesicular rash appeared on her extremities and thorax. Clinical laboratory results showed thrombocytopenia (100 × 10^3^ platelets/mL [reference range 140–440 × 10^3^ platelets/mL]) with slightly prolonged clotting times of thrombin and prothrombin, and neutrophilia.

Rickettsiosis was diagnosed on the basis of PCR amplification and sequencing of bacterial genes; immunofluorescent assay (IFA) and restriction fragment length polymorphism (RFLP) analyses confirmed the diagnosis and identified the *Rickettsia* species. Blood was collected in 3.8% sodium citrate as anticoagulant, and DNA was extracted immediately by QIAamp DNA kit (QIAGEN, Valencia, CA, USA) following the manufacturer’s instructions. PCR amplification was performed by using genus-specific primers for the rickettsial 17-kDa protein gene (5′-GCTCTTGCAACTTCTATGTT-3′ and 5′-CATTGTTCGTCAGGTTGGCG-3′) (434-bp PCR fragment) and the outer membrane protein B (*omp*B) primers (5′-ATGGCTCAAAAACCAAATTTTCTAA-3′ and 5′-GCTCTACCTGCTCCATTATCTGTACC-3′) (996-bp PCR fragment). The positive controls used were DNA of *R. felis*, *R. rickettsii*, *R. akari*, *R. typhi*, *R. conorii*, and *R. honei*, provided by the Rickettsial and Ehrlichial Diseases Research Laboratory (University of Texas Medical Branch, Galveston, TX, USA); 1 reaction without DNA was used as a negative control. To avoid contamination, DNA of the positive controls and the patients was handled separately.

PCR products from the 17-kDa PCR were digested with the *Alu*I (Invitrogen, Carlsbad, CA, USA) restriction enzyme (Figure). Sequences of the *omp*B PCR and 17-kDa products were compared at the National Center for Biotechnology Information by using the BLAST software (*12*). The *omp*B sequence showed 100% identity with *R. akari* (GenBank accession no. CP000847), 92% with *R. australis* (GenBank accession no. AF123709.1), 90% with *R. felis* (GenBank accession no. CP000053.1), and <90% with other *Rickettsia* spp. The 17-kDa sequence showed 100% identity with *R. akari* (GenBank accession no. CP000847.1), and <95% with *R. rickettsii* (GenBank accession no. CP000848.1), *R. conorii* (GenBank accession no. AE006914.1), and other *Rickettsia* spp.

**Figure:**
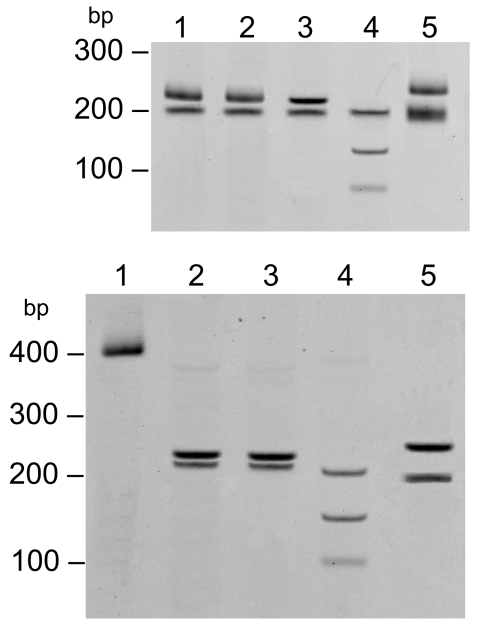
Restriction fragment length polymorphism of the 17-kDa PCR product (434 bp) digested with *Alu*I. Top: lane 1, 32-year-old woman; lane 2, 9-year-old girl; lane 3, *Rickettsia akari*–positive control; lane 4, *R. rickettsii*–positive control; lane 5, *R. typhi*–positive control. Bottom: lane 1, undigested 17-kDa gene PCR amplicon; lane 2, 9-year-old girl; lane 3, 32-year-old woman; lane 4, *R. conorii*–positive control; lane 5, *R. honei–*positive control.

IFA showed that both patients had moderate antibody titers reactive with *R. akari* (patient 1, 256; patient 2, 128), low antibody titers reactive with *R. rickettsii* (patient 1, 64; patient 2, 32), and no antibodies to *R. typhi* antigens. A positive human serum control and IFA slides were provided by the Rickettsial and Ehrlichial Diseases Research Laboratory. We detected immunoglobulin M by using a μ heavy chain–specific conjugate. Only an acute serum sample was collected from each patient during the 12–21 days after illness onset. Positive serum samples were serially diluted to 1:4,096 to determine the endpoint titer.

Patient 1 was treated with intravenous chloramphenicol, 75 mg/kg 1× per day for 7 days; clinical signs were reduced in 36 hours. Both patients were treated with 100 mg of oral doxycycline 2× per day for 7 days; symptoms were reduced in 48–72 hours.

## Conclusions

Human rickettsioses have tremendously affected public health in the Americas. In the past decade, several Latin American countries have reported infected vectors, potential reservoirs, and human cases of rickettsial infections. Human cases have been limited to infections with *R. rickettsii*, *R. felis*, *R. prowasekii*, *R. typhi,* and *R. parkeri* (*6**–**11*). In southeastern Mexico, *R. rickettsii* and *R. felis* have been the only rickettsiae detected for many years; several cases of human illness and even deaths have occurred (*6**,**8*).

Rickettsialpox is a benign, self-limiting disease that usually resolves within 14–21 days; no deaths from rickettsialpox have been reported. However, for 1 of our patients, hemorrhage was the most prominent sign; the severity of the clinical features could have caused death had the correct treatment not been instituted promptly. Although hemorrhages have not been described for rickettsialpox, dissimilarities in the clinical features of rickettsial disease among countries have been reported (*13*). Organ infections caused by increased vascular permeability have been described in rickettsial infections with different degrees of severity. Rickettsialpox has even been associated with hepatitis, a not well-documented complication of rickettsialpox (*14*).

The similarity among symptoms of rickettsial infections and other febrile illnesses endemic to Yucatan, such as dengue fever, and the continuous environmental exposures of rural inhabitants to vectors of rickettsial diseases encouraged us to implement epidemiologic surveillance. Our 2 patients were detected by this surveillance. Both patients came from the relatively close rural areas of Yucatan and Campeche, Mexico, and reported mice near their homes.

Although our patients did not have eschars, we diagnosed the infection as *R. akari* serologically and molecularly. However, if IFA gives inconclusive results, PCR amplification of the bacterial genome is the decisive parameter for diagnosis. Our report of these 2 human rickettsialpox cases in Mexico provides a new rickettsial infection to consider in the differential diagnosis of febrile illnesses.
